# Assessment of the ergonomic risk from saddle and conventional seats in dentistry: A systematic review and meta-analysis

**DOI:** 10.1371/journal.pone.0208900

**Published:** 2018-12-17

**Authors:** Giovana Renata Gouvêa, Walbert de Andrade Vieira, Luiz Renato Paranhos, Ítalo de Macedo Bernardino, Jaqueline Vilela Bulgareli, Antonio Carlos Pereira

**Affiliations:** 1 Department of Community Dentistry, School of Dentistry of Piracicaba, University of Campinas, Piracicaba, SP, Brazil; 2 Department of Dentistry, University of Sergipe, Aracaju, SE, Brazil; 3 Department of Preventive and Community Dentistry, School of Dentistry, Federal University of Uberlândia, Uberlândia, MG, Brazil; 4 Postgraduate Program in Dentistry, State University of Paraíba, Campina Grande, PB, Brazil; Eberhard-Karls-Universitat Tubingen Medizinische Fakultat, GERMANY

## Abstract

**Objective:**

This study aimed to verify whether the saddle seat provides lower ergonomic risk than conventional seats in dentistry.

**Methods:**

This review followed the PRISMA statement and a protocol was created and registered in PROSPERO (CRD42017074918). Six electronic databases were searched as primary study sources. The "grey literature" was included to prevent selection and publication biases. The risk of bias among the studies included was assessed with the Joanna Briggs Institute Critical Appraisal Tool for Systematic Reviews. Meta-analysis was performed to estimate the effect of seat type on the ergonomic risk score in dentistry. The heterogeneity among studies was assessed using I^2^ statistics.

**Results:**

The search resulted in 3147 records, from which two were considered eligible for this review. Both studies were conducted with a total of 150 second-year dental students who were starting their laboratory activities using phantom heads. Saddle seats were associated with a significantly lower ergonomic risk than conventional seats [right side (mean difference = -3.18; 95% CI = -4.96, -1.40; p < 0.001) and left side (mean difference = -3.12; 95% CI = -4.56, -1.68; p < 0.001)], indicating posture improvement.

**Conclusion:**

The two eligible studies for this review provide moderate evidence that saddle seats provided lower ergonomic risk than conventional seats in the examined population of dental students.

## Introduction

Occupational health has been extensively investigated in dentistry [1–4], considering that dentists are professionals highly vulnerable to musculoskeletal diseases [5,6], especially in the cervical and lumbar spines [7]. Working posture is the main risk factor for developing musculoskeletal disorders [8–9].

The sitting posture is the body position that dentists use most frequently [10]. The dental stool has an influence on such posture [11–13], because it induces the use of certain postural patterns to find a more comfortable and/or functional position [11–13]. In addition, the curvature of the spine, as well as the location and correct position of the head and pelvis are crucial for the biomechanics of the sitting position [14–16].

There is evidence that the 90° sitting posture (knee angle and hip angle) increases the passive tension of hamstring muscles, causing a posterior pelvic rotation and resulting in a kyphotic sitting posture of the lumbar spine [17–18]. However, ergonomic recommendations [19], radiographic studies [17–18], and analyses from physical therapists [20] and laypersons [21,22] indicate that a sitting posture with a slight anterior tilt of the lumbar spine and a slight lumbar lordosis of the lumbar spine reduces the incidence of low back pain most efficiently.

Aiming to reduce postural problems in dentistry, scientific studies have been performed to elucidate the impact of different types of seats on the posture of students and trained professionals [16,23], as well as the importance of ergonomic seat interventions [14] in reducing musculoskeletal symptoms [15]. However, the literature does not yet provide a consensus on whether the saddle seat is a superior alternative to the conventional seat for maintaining optimal posture.

Thus, the present study aimed to answer the following guiding question (based on the PICO strategy): “Does the saddle seat (intervention) provide lower ergonomic risk (outcome) to dentists and/or dental students (population) when compared with conventional seats (comparison)?” The authors have tested the hypothesis that using the saddle seat will promote lower ergonomic risk than the conventional seat.

## Methods

### Protocol and registry

This systematic review was performed following the PRISMA (S1 PRISMA Checklist) statement (Preferred Reporting Items for Systematic Reviews and Meta-Analyses) [24] and the Cochrane guidelines [25]. The systematic review protocol was registered in the PROSPERO database under number CRD42017074918 (https://www.crd.york.ac.uk/PROSPERO/).

### Study design and eligibility criteria

The review included only randomized controlled trials that compared the working posture of dental students and/or dentists in conventional seats without ergonomic changes and in ergonomic saddle seats. There were no restrictions of year, language, or publication status (ahead of print).

The following were excluded: 1) Studies not related to the topic; 2) Reviews, letters to the editor, personal opinions, book/book chapters, didactic material, reports, abstracts, and patents; 3) Qualitative or prevalence studies; and 4) Studies that used other types of seats or modified seats.

### Sources of information and research

The primary sources of research were the electronic databases Embase, Latin American and Caribbean Health Sciences (LILACS), PubMed (including MedLine), SciELO, Scopus, and Web of Science. OpenThesis and OpenGrey were used to collect the “grey literature”, avoiding selection and publication biases. A manual search was also performed through a systematic analysis of the references of the eligible articles.

Two eligibility reviewers conducted the research independently (GG and WAV). The DeCS (Descriptors in Health Sciences– http://decs.bvs.br) and MeSH (Medical Subject Headings– https://www.ncbi.nml.nih.gov/mesh) resources were used for keyword selection. The Boolean operators “AND” and “OR” were applied to enhance the search strategy through several combinations (S1 Table). The bibliographical research was developed and performed in August 2017. The search strategy included the following MeSH, DeCS, and Emtree terms: ‘Dentists’, ‘Posture’, ‘Human Engineering’, ‘Odontologia’ [Portuguese], ‘Postura’ [Portuguese] associated with the entry terms: ‘Dental students’, ‘Student of dentistry’, ‘Undergraduate student of dentistry’, ‘Seated Position’, ‘Sitting Position’, ‘Saddle chair’, ‘Saddle seat’. The records obtained were exported to the software EndNote Basic/Online, desktop version (Thomson Reuters, New York, USA) and duplicates were removed.

### Selection of studies

The studies were selected in three stages. In stage 1, two reviewers (GG and WAV) performed a systematic analysis of the titles, independently. The articles whose titles met the objectives of the study were selected for stage 2, when both reviewers (GG and WAV) also performed a systematic analysis of the abstracts. At this time, the studies not related to the topic, reviews, letters to the editor, personal opinions, book/book chapters, didactic material, reports, abstracts, patents, qualitative or observational studies, and studies that used other types of seats or modified ones were excluded. The articles whose titles met the study objectives, but had no abstract, were fully reviewed.

In the third stage, the full texts of the preliminary eligible studies were obtained and evaluated to verify whether they met the eligibility criteria. When both reviewers could not reach an agreement, a third reviewer (LRP) was consulted to make a final decision. Rejected studies were recorded separately along with the explicit reasons for exclusion.

### Process of data collection and extraction

After the selection, two authors (MSS and WAV) analyzed the studies, which data were extracted for the following information: article identification (author, year, study location), sample characteristics (number of patients in each study, mean age, sex distribution, school year), type of intervention (seat type, training time, evaluation start time), and methods for obtaining the results (methods used for posture evaluation, image analysis, and calibration time). Any disagreement was discussed and a third reviewer (LRP) was consulted when necessary.

### Individual risk of bias of the studies

The risk of bias in the studies selected was assessed using the Joanna Briggs Institute Critical Appraisal tools for use in JBI Systematic Reviews for Randomized Controlled Trials [26]. Two authors (WAV and LRP) independently assessed each domain for the potential risk of bias. The following questions were used for the assessment: 1) Was true randomization used for assigning the participants to treatment groups? 2) Was the allocation to treatment groups concealed? 3) Were treatment groups similar at baseline? 4) Were participants blind to treatment assignment? 5) Were those delivering treatment blind to treatment assignment? 6) Were outcome assessors blind to treatment assignment? 7) Were treatment groups treated identically other than the intervention of interest? 8) Was follow-up complete, and if not, were differences between groups in terms of their follow-up adequately described and analyzed? 9) Were participants analyzed in the groups to which they were randomized? 10) Were outcomes measured in the same way for treatment groups? 11) Were outcomes measured in a reliable way? 12) Was appropriate statistical analysis used? 13) Was the trial design appropriate, and were any deviations from the standard RCT design (individual randomization, parallel groups) accounted for in the conduct and analysis of the trial? The risk of bias was categorized as High when the studies reached up to 49% of “yes” score, Moderate when they reached 50% to 69% of “yes” score, and Low when the studies reached more than 70% of “yes” score. Studies categorized as either high risk of bias or low methodological quality were eliminated.

### Outcome measures and data analysis

The meta-analysis for continuous outcome was performed to estimate the effect of seat type on the ergonomic risk score in dentistry [25]. The mean difference was used for pooling effects. Heterogeneity among studies was assessed using I^2^ statistics and classified as follows: low (I^2^ < 25%), moderate (I^2^ = 50%), and high (I^2^ > 75%) [27]. The random-effects model was selected to minimize the effect of heterogeneity among studies [28]. Publication bias was not assessed because there was not a sufficient number of studies to group in a funnel plot. The software Review Manager, version 5.3 (RevMan, Cochrane Collaboration) was used to perform all statistical analyses.

### Confidence in cumulative evidence

The Grading of Recommendation, Assessment, Development, and Evaluation (GRADE) tool [28] assessed evidence quality and grading of recommendation strength. This assessment was based on study design, methodological limitations, inconsistency, indirectness, imprecision, and other considerations. Evidence quality was characterized as high, moderate, low, or very low [29].

## Results

### Selection of studies

**Fig 1:**
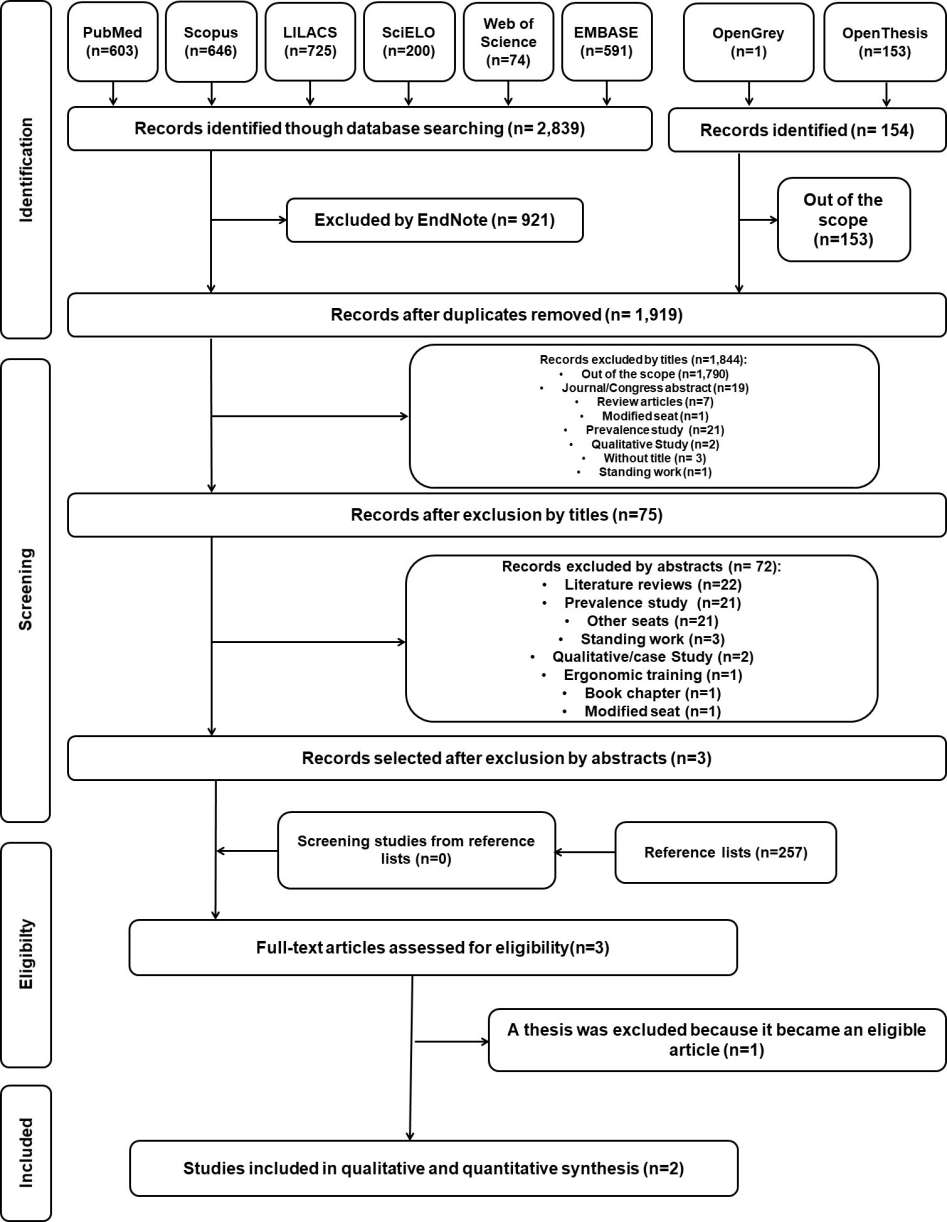
Flowchart of the process of searching and selecting the literature, adapted from the PRISMA statement.

The bibliographical research was developed and performed in August 2017. During the first stage of study selections, 2993 records were found in six electronic databases. After removing the repeated/duplicated records, 1918 articles proceeded to the analysis of titles and abstracts. A total of 154 studies from the “grey literature” was found through the search strategy, although only one was related to the objectives of the present review. After the analysis of titles and abstracts, only three studies were eligible for full-text analysis. The references of the initially eligible studies were carefully assessed to verify potential articles that were absent from the main search strategy. However, from the three studies included in this stage, one of them was excluded for being a thesis from which an eligible article was produced. Therefore, two articles proceeded to the analysis of results. Fig 1 reproduces the process of search, identification, inclusion, and exclusion of articles.

### Characteristics of the studies

Both eligible studies [11–12] commented on the research ethical criteria and explained the use of consent forms for research subjects. None of the studies presented either sample calculation or study power. The analysis resulted in a total sample of 150 dental students and there were no studies with professional dentists. The studies were performed in the United Kingdom [11] in 2007 and in India [12] in 2014. One study compared the Salli Saddle Chair and a conventional chair with and without back rest and flat surface [12], and the other compared a Bambach Saddle Seat and a conventional chair with back rest and flat surface [11]. Both studies [11–12] were performed with second-year dental students, who were starting their laboratory activities using phantom heads.

The participants of the eligible studies [11–12] received training as to correct posture and use of each seat type. The evaluation was performed after 10 [11] or 12 [12] weeks so the students would get used to the seats. Table 1 presents a summary of the main characteristics of these studies.

**Table 1:** Summary of the main characteristics of the eligible studies.

Author, year, and country	Seat type	Sample (n)	School period	Location	Procedure performed	Training time	Time of assessment	Evaluation method	Analysis method	Calibration time
*Gandavadi et al*., *2007*,*United Kingdom*	Bambach Saddle Seat (BSS) Conventional Seat (CS)	Bambach Saddle Seat: 30 Conventional Seat: 30	2^nd^ year	Preclinical laboratory	Cavity preparation of mandibular teeth in a mannequin	10 weeks	2 weeks	RULA*	Photos	10 minutes
*Dable et al*.,*2014*, *India*	Salli Saddle Chair (SSC) Conventional chair with back rest (CC1) Conventional chair without back rest (CC2)	Salli Saddle Chair: 30 Conventional chair with back rest: 30 Conventional chair without back rest: 30	2^nd^ year	Preclinical laboratory	Cavity preparation of the first mandibular premolar in a mannequin	12 weeks	3 days	RULA*	Videos	15 minutes

*RULA: Rapid Upper Limb Assessment.

### Risk of bias in the studies

Both studies included in this review [11–12] presented low risk of bias in the Joanna Briggs Institute Critical Appraisal tool [26]. Table 2 shows detailed information on the risk of bias of the studies included.

**Table 2:** Risk of bias assessed by the Joanna Briggs Institute Critical Appraisal Tools for use in JBI Systematic Reviews for Randomized Controlled Trials” [26].

Authors	Q.1	Q.2	Q.3	Q.4	Q.5	Q.6	Q.7	Q.8	Q.9	Q.10	Q.11	Q.12	Q.13	%yes/risk
*Gandavadi et al*., *2007*	√	√	√	—	√	—	√	√	√	√	√	√	√	84.6%/Low
*Dable et al*., *2014*	√	√	√	—	√	—	√	√	√	√	√	√	√	84.6%/Low

1) Was true randomization used for assigning the participants to treatment groups? 2) Was the allocation to treatment groups concealed? 3) Were treatment groups similar at baseline? 4) Were participants blind to treatment assignment? 5) Were those delivering treatment blind to treatment assignment? 6) Were outcome assessors blind to treatment assignment? 7) Were treatment groups treated identically other than the intervention of interest? 8) Was follow-up complete, and if not, were differences between groups in terms of their follow-up adequately described and analyzed? 9) Were participants analyzed in the groups to which they were randomized? 10) Were outcomes measured in the same way for treatment groups? 11) Were outcomes measured in a reliable way? 12) Was appropriate statistical analysis used? 13) Was the trial design appropriate, and were any deviations from the standard RCT design (individual randomization, parallel groups) accounted for in the conduct and analysis of the trial? NA = Not Applicable; √ = Yes; “–” = No.

### Results of individual studies and meta-analysis

The studies selected used the RULA (Rapid Upper Limb Assessment) method [30], which analyzes the overload concentrated in the neck and upper limbs during work and assesses the static muscle work and the forces exerted by the segments analyzed. The calibration time set by the studies ranged from 10 [11] to 15 [12] minutes so that the students could focus on their work and be evaluated afterwards. In both studies, the students prepared a mandibular tooth in a mannequin.

In the study by Gandavadi *et al*. [11], photographs were taken of both left and right sides, while in the study by Dable *et al*. [12], the analysis was performed from static images captured from videos. The results showed lower scores for the ergonomic seats (Salli Saddle Chair and Bambach Saddle Seat) than for conventional seats. In the study by Dable *et al*. [12], the authors also used image magnification lenses to compare the groups, showing even lower scores with such system.

**Fig 2:**
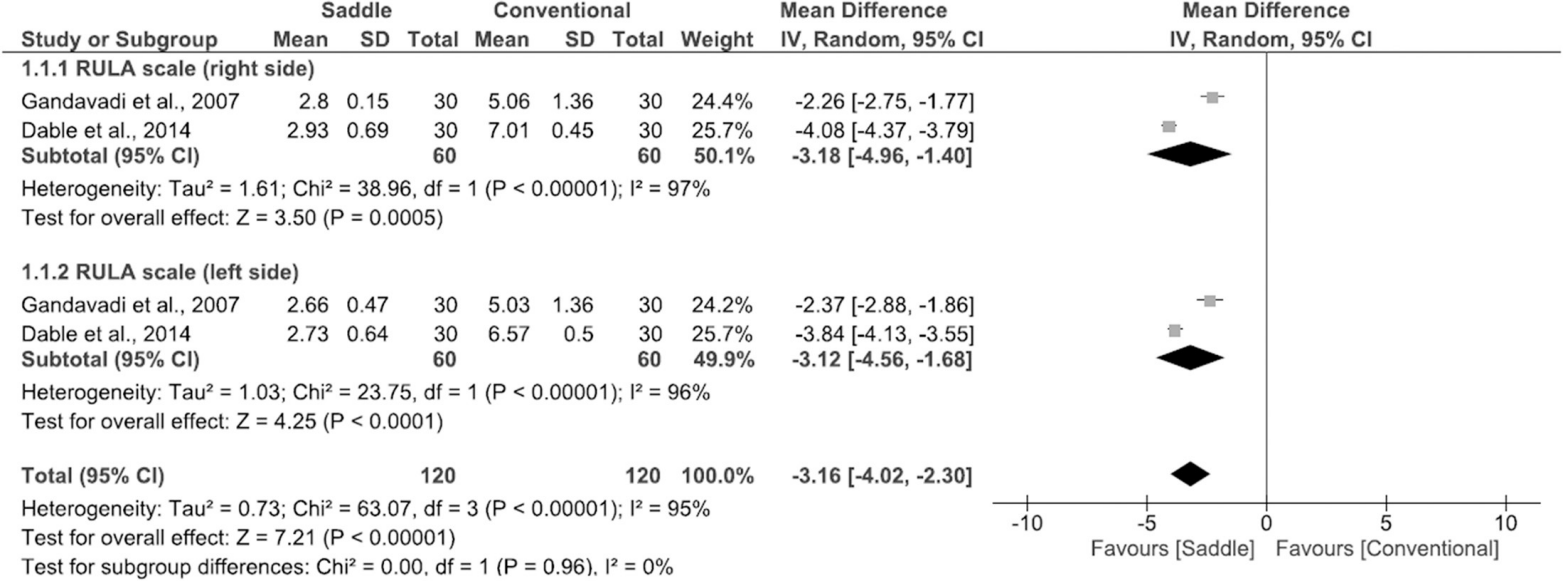
Effect of seat type (saddle versus conventional) on ergonomic risk score in dentistry, assessed using the RULA scale.

Fig 2 presents the forest plots. The mean differences in ergonomic risk score and their respective 95% confidence intervals are represented by squares for the individual studies. The S2 Table shows the risk score of individual studies. The diamonds at the bottom represent the pooled mean ergonomic risk score with 95% confidence interval. The meta-analysis results showed that saddle seats are associated with significantly lower ergonomic risk scores when compared with conventional seats [right side (mean difference = -3.18; 95% CI = -4.96, -1.40; p < 0.001) and left side (mean difference = -3.12; 95% CI = -4.56, -1.68; p < 0.001)], indicating posture improvement. The overall mean difference in ergonomic risk score was -3.16 (95% CI = -4.02, -2.30; p <0.001). Between-study heterogeneity was high (I^2^ = 95%; p <0.001).

### Confidence in cumulative evidence

Overall, the quality of evidence from the outcomes evaluated by the GRADE system [29] was assessed as moderate (Table 3).

**Table 3:** Grading of Recommendation, Assessment, Development, and Evaluation (GRADE) summary of results table for the outcomes of the systematic review and meta-analysis [29].

Quality Assessment							Summary of Results			Importance
Number of studies	Study Design	Methodological Limitations	Inconsistency	Indirectness	Imprecision	Publication Biases	Number of participants		General Quality	
							Intervention	Comparison		
**2**	Randomized controlled trials	X^1^	√	√	√	√	60	60	+++ MODERATE	Critical

GRADE factors: = √, no serious limitations; X, serious limitation. General quality of evidence: +, very low; ++, low; +++, moderate; ++++, high.

^1^ Absence of blinding of outcome assessors and participants.

## Discussion

This study aimed to compare the ergonomic risk of saddle and conventional seats used in work practices of dentists and/or dental students. Both eligible studies [11–12] were performed with a convenience sample (dental students). Studies with trained professionals may result in bias due to the different situations of the clinical routine. Forming a control group for this type of study, paired with the experimental group for age and time of profession, would represent another challenge. These variables may reflect especially in existing musculoskeletal diseases and in the resistance for changing usual postural practices [31–32]. Thus, the results of the present meta-analysis with studies performed with dental students significantly favor saddle seats over conventional seats, which confirms the initial hypothesis.

In both eligible studies [11–12], dental students were instructed to prepare a cavity in the mandibular teeth of a mannequin, at the preclinical laboratory. It is known that a procedure performed in a dental mannequin does not reproduce the actual reality of a dentist’s routine. This is because a real patient presents variables such as age (elderly people or children), anatomical structures (tongue, cheek, and mouth opening limitation), special care (physical and/or mental disabilities), altered psychological states (fear and/or anxiety), obesity, and pregnancy, which may change and complicate the operational procedure. However, in the preclinical laboratory during procedures in mannequins, students experience the first body postures, adapting their body to seat, static posture, reduced field of vision, dental procedure, precision of fine movements, and especially to the fear and insecurity of dealing with something new [33].

One of the methods for verifying ergonomic risks is the Rapid Upper Limb Assessment (RULA) [30], which is the most cited in the literature and used in both eligible studies [11–12] of this review. In this method, the positions of individual body segments are observed and assessed with increasing scores according to the growing deviation of the neutral posture [30]. Different studies [30–34] have assessed the validity and reliability of the RULA, which is considered an adequate method to assess the body posture of dentists [35] and dental students [36]. The observations of evaluators regarding the static image may be associated with the uncertainty regarding camera angle [37].

Gandavadi *et al*. [11] observed the working postures of both right and left sides using digital photographs. Dable *et al*. [12], in turn, used videos that were paused at every postural position and at every body movement of both right and left sides. However, the assessment and final score of both studies [11–12] were based on a static image. The assessment of the body posture images of the research participants started after 10 to 15 minutes in a familiar environment. Given the long time for capturing the images, the participants were likely focused on the activity proposed and kept the postural habits of their usual routine, which canceled the Hawthorne effect [38]—a phenomenon in which participants change their behavior when they are aware of being watched.

In this study, the ergonomic risk was assessed in groups that used conventional and saddle seats. The results indicated an intermediate to high score for ergonomic risk in the group using the conventional seat, which is consistent with other studies [36,39]. Over the last decade, research has been intensified, designing the effects of different seats on the clinical practice of dentists and dental students [11–13,40–42]. Among such studies, three have investigated the ergonomically modified stool [40–42] and three have investigated the saddle seat [11–13]. All studies showed an improvement in the experimental group when compared to the control group, especially for presenting a lumbar lordosis seated posture [11–13].

There is a consensus among several studies [17–21,43–49] that the lumbar lordosed seated posture is optimal for favoring a neutral lumbar posture, minimizing the painful symptomatology of low back pain. It is also associated with high muscular activity and the increase in spinal load due to the posterior pelvic tilt, which is then balanced by muscle contractions in the dorsal spine, representing a dynamic posture [50]. This posture is obtained by positioning the lower lumbar spine in a slight forward tilt and slight lumbar lordosis, while maintaining the relaxation of the muscles surrounding the thoracic spine [20].

In occupational science, a static body posture is defined as a posture held for more than four seconds [51–52]. Static work procedures prevent the blood flow required for tissue recovery. Other significant factors are the frequency of occurrence, the pauses during movement, and the duration (time component) for maintaining a static body posture [53]. Consequently, several dental tasks are performed in static postures with the prolonged flexion and/or rotation of the trunk, presenting a potential risk for the musculoskeletal system [53].

The interdisciplinarity between bioengineering and health sciences improves clinical relevance and research [19,54–56]. Dynamic seats [57–58] with a slight forward inclination [54,56–59], with or without a low backrest [57] to support the ischia [19,60], are the challenges of novel seat designs. However, it is worth noting that adopting a good posture and using the correct furniture are not enough to reduce the overload on the osteomyoarticular tissues of dentists [61–62]. Besides seat design, the human, occupational, and organizational factors also play an important role in terms of load conditions in the human body [63–65]. Psychosocial factors are also major risk factors for persistent low back pain in workers, and they should be considered along with the physical labor requirements, reducing the disability related to lumbar pain [66]. Such pain is also directly associated with depression and somatization [67]. Psychosocial interventions may reduce the impact of low back pain in the workplace [68,69].

Four-handed dentistry, equipment organization in the workspace, correct positioning of patients, illumination, and auxiliary components should be observed and controlled in the dental clinical practice [61–62]. The musculoskeletal stress of a dental professional is quantifiable, comparable, and especially rather variable, considering that musculoskeletal disorders may be reduced by improving the ergonomic positioning of the patient and the practitioner [70]. Positioning should maintain the natural curves of the lumbar spine (cervical lordosis, thoracic kyphosis, lumbar lordosis, and sacral kyphosis), allowing a neutral sitting posture [7,59] so that muscles and intervertebral discs may alternate between relaxation and loading. Correct positioning is beneficial for nourishing muscles [71] and intervertebral discs [72] and for potentially reducing ergonomic risks.

The present review is original, and it has contributed to develop the scientific knowledge from two main points. Primarily, it is the first systematic literature review to investigate the influence of seat type on ergonomic risk among dental students. Second, the low risk of bias observed in the eligible studies allows drawing more consistent and reliable conclusions from the data obtained, producing major implications for the academic dental clinical practice.

### Limitations

The present study is limited by the presence of only two clinical studies on the subject, with no sample calculation or study power. In addition, the student population included only dental students working on phantom heads and it was not sex-specific. In both studies included, the data were collected only at the end of follow-up. It is worth noting that short-term investigations of the sitting posture may not completely represent the biological time-dependent responses. Further studies should be performed to determine whether the effectiveness of a saddle seat intervention is maintained in the long-term, especially concerning the neutral lumbar posture. In addition, both eligible articles used static images to represent the average posture of a person, which does not fit the reality. Therefore, further studies need to employ state of the art posture measurement equipment that automatically record the posture continuously. A combination of posture and Electromyography (EMG) measurement would provide additional insight.

## Conclusion

The two eligible studies for this review provide moderate evidence that saddle seats provided lower ergonomic risk than conventional seats in the examined population of dental students. Follow-up studies are required to confirm this result by addressing the limitations of the studies. For example, follow-up studies should employ state of the art posture measurement equipment and examine whether saddle seats also provide lower ergonomic risk in a population of professional dentists treating real patients.

## Supporting information

S1 PRISMA Checklist PRISMA checklist. (DOC)

S1 Table Strategies for database search. (DOCX)

S2 Table Main results of eligible articles. (DOCX)
