# Supplementary material for: Assessment of the ergonomic risk from saddle and conventional seats in dentistry: A systematic review and meta-analysis
Source: PLoS One. 2018 Dec 17;13(12):e0208900. doi: 10.1371/journal.pone.0208900 (PMC6296655; doi:10.1371/journal.pone.0208900)
Supplement: S1 Table — (DOCX) [file pone.0208900.s002.docx]

**Supplementary file -** Strategies for database search.

| Database | Search Strategy (August, 2017) |  |
| --- | --- | --- |
| PubMed  http://www.ncbi.nlm.nih.gov/pubmed | ("dentists"[MeSH Terms] OR "dentists"[All Fields] OR "dental students"[All Fields] OR "student of dentistry"[All Fields] OR "undergraduate student of dentistry"[All Fields]) AND ("Posture"[MeSH Terms] OR "Posture"[All Fields] OR "human engineering"[MeSH Terms] OR "human engineering"[All Fields] OR "Seated Position"[All Fields] OR "Sitting Position"[All Fields] OR "Saddle chair"[All Fields] OR "Saddle seat"[All Fields]) | 603 |
| Web Of Science  http://apps.webofknowledge.com/ | ((("dentists" OR "dental students" OR "student of dentistry" OR "undergraduate student of dentistry") AND ("posture" OR “human engineering” OR "seated position" OR "sitting position" OR "saddle chair" OR "saddle seat"))) | 74 |
| Embase  http://www.embase.com/ | ('dentists'/exp OR 'dentists' OR 'dental students'/exp OR 'dental students' OR 'student of dentistry' OR 'undergraduate student of dentistry') AND ('posture'/exp OR 'posture' OR 'human engineering'/exp OR 'human engineering' OR 'seated position' OR 'sitting position'/exp OR 'sitting position' OR 'saddle chair' OR 'saddle seat') | 591 |
| Scopus  http://www.scopus.com/ | ((("dentists" OR "dental students" OR "student of dentistry" OR "undergraduate student of dentistry" ) AND ( "posture" OR "human engineering" OR "seated position" OR "sitting position" OR "saddle chair" OR "saddle seat"))) | 646 |
| LILACS  http://lilacs.bvsalud.org/ | (((("dentists" OR "dentistry" OR "dental students" OR "student of dentistry" OR "undergraduate student of dentistry") AND ("Posture" OR "Seated Position" OR "Sitting Position" OR "human engineering" OR "Dental Equipment" OR "Equipment Dental" OR "Saddle chair" OR "Saddle seat"))) AND (instance:"regional") AND ( db:("LILACS"))) AND (instance:"regional") AND ( db:("LILACS")) | 160 |
|  | (“cirurgião-dentista” AND "postura") AND (instance:"regional") AND ( db:("LILACS")) | 21 |
|  | (“cirurgião-dentista” AND "postura sentada" ) AND (instance:"regional") AND ( db:("LILACS")) | 7 |
|  | (“cirurgião-dentista” AND “mocho odontológico” ) AND (instance:"regional") AND ( db:("LILACS")) | 2 |
|  | (“cirurgião-dentista” AND “Assento tipo sela”) AND (instance:"regional") AND ( db:("LILACS")) | 0 |
|  | (“estudantes de odontologia” AND "postura" ) AND (instance:"regional") AND ( db:("LILACS")) | 17 |
|  | (“estudantes de odontologia” AND "posição sentada") AND (instance:"regional") AND ( db:("LILACS")) | 0 |
|  | (“estudantes de odontologia” AND “mocho odontológico”) AND (instance:"regional") AND ( db:("LILACS")) | 0 |
|  | (“estudantes de odontologia” AND “Assento tipo sela”) AND (instance:"regional") AND ( db:("LILACS")) | 0 |
|  | (“odontologia” AND "postura" ) AND (instance:"regional") AND ( db:("LILACS")) | 88 |
|  | (“odontologia” AND "postura sentada" ) AND (instance:"regional") AND ( db:("LILACS")) | 25 |
|  | (“odontologia” AND “mocho odontológico” ) AND (instance:"regional") AND ( db:("LILACS")) | 2 |
|  | (“odontologia” AND “assent tipo sela” ) AND (instance:"regional") AND ( db:("LILACS")) | 0 |
|  | (“dentista” AND "postura" ) AND (instance:"regional") AND ( db:("LILACS")) | 27 |
|  | (“dentista” AND "postura de sentado" ) AND (instance:"regional") AND ( db:("LILACS")) | 8 |
|  | (“dentista” AND “equipo dental” ) AND (instance:"regional") AND ( db:("LILACS")) | 31 |
|  | (“dentista” AND “"Taburete de silla de montar"”) AND (instance:"regional") AND ( db:("LILACS")) | 0 |
|  | (“estudiantes de odontología” AND "postura" ) AND (instance:"regional") AND ( db:("LILACS")) | 9 |
|  | (“estudiantes de odontología” AND "posição de sentado") AND (instance:"regional") AND ( db:("LILACS")) | 0 |
|  | (“estudiantes de odontología” AND “equipo dental” ) AND (instance:"regional") AND ( db:("LILACS")) | 20 |
|  | (“estudiantes de odontología” AND “Taburete de silla de montar”) AND (instance:"regional") AND ( db:("LILACS")) | 0 |
|  | (“odontología” AND "postura" ) AND (instance:"regional") AND ( db:("LILACS")) | 88 |
|  | (“odontología” AND "posição de sentado") AND (instance:"regional") AND ( db:("LILACS")) | 0 |
|  | (“odontología” AND “equipo dental”) AND (instance:"regional") AND ( db:("LILACS")) | 220 |
|  | (“odontología” AND “Taburete de silla de montar”) AND (instance:"regional") AND ( db:("LILACS")) | 0 |
| SciELO  http://www.scielo.org/ | (("dentists" OR "dental students" OR "student of dentistry" OR "undergraduate student of dentistry") AND ("posture" OR human engineering OR "seated position" OR "sitting position" OR "saddle chair" OR "saddle seat")) | 107 |
|  | cirurgião-dentista AND postura | 3 |
|  | cirurgião-dentista AND postura sentada | 0 |
|  | cirurgião-dentista AND mocho odontológico | 0 |
|  | cirurgião-dentista AND assento tipo sela | 0 |
|  | estudantes de odontologia AND postura | 4 |
|  | estudantes de odontologia AND posição sentada | 0 |
|  | estudantes de Odontologia AND mocho odontológico | 0 |
|  | estudantes de Odontologia AND assento tipo sela | 0 |
|  | odontologia AND postura | 14 |
|  | odontologia AND posição sentada | 0 |
|  | odontologia AND mocho odontológico | 0 |
|  | odontologia AND assento tipo sela | 0 |
|  | dentista AND postura | 6 |
|  | dentista AND postura de sentado | 0 |
|  | dentista AND equipo dental | 10 |
|  | dentista AND Taburete de silla de montar | 0 |
|  | estudiantes de Odontología AND postura | 10 |
|  | estudiantes de Odontología AND posição de sentado | 0 |
|  | estudiantes de Odontología AND equipo dental | 6 |
|  | estudiantes de Odontología AND taburete de silla de montar | 0 |
|  | odontología AND postura | 14 |
|  | odontología AND posição de sentado | 0 |
|  | odontología AND equipo dental | 26 |
|  | odontología AND taburete de silla de montar | 0 |
| OpenGrey  http://www.opengrey.eu/ | (("dentists" OR "dental students" OR "student of dentistry" OR "undergraduate student of dentistry") AND ("posture" OR “human engineering” OR "seated position" OR "sitting position" OR "saddle chair" OR "saddle seat")) | 1 |
| OpenThesis  http://www.openthesis.org/ | (("dentists" OR "dental students" OR "student of dentistry" OR "undergraduate student of dentistry") AND ("posture" OR “human engineering” OR "seated position" OR "sitting position" OR "saddle chair" OR "saddle seat")) | 153 |
| TOTAL | | **2993** |
