# Supplementary material for: Assessment of the ergonomic risk from saddle and conventional seats in dentistry: A systematic review and meta-analysis
Source: PLoS One. 2018 Dec 17;13(12):e0208900. doi: 10.1371/journal.pone.0208900 (PMC6296655; doi:10.1371/journal.pone.0208900)
Supplement: S2 Table — (DOCX) [file pone.0208900.s003.docx]

**S2 Table** - Main results of the eligible articles.

| Author | RULA Score: Right side (mean) | RULA Score: Left side  (mean) | RULA Score with magnified image |
| --- | --- | --- | --- |
| *Gandavadi et al., 2007* | Bambach Saddle Seat: 2.80  Conventional Seat: 5.06 | Bambach Saddle Seat: 2.66  Conventional Seat: 5.03 | ** |
| *Dable et al., 2014* | Salli Saddle Chair: 2.93 ± 0.69  Conventional chair with back rest: 7.01 ± 0.45  Conventional chair without back rest: 7.03 ± 0.49 | Salli Saddle Chair: 2.73 ± 0.64  Conventional chair with back rest: 6.57 ± 0.50  Conventional chair without back rest: 6.96 ± 0.56 | Salli Saddle Chair: 1.57 ± 0.50  Conventional chair with back rest: 5.63 ± 0.49  Conventional chair without back rest: 5.07 ± 0.46 |

** Not mentioned by the author; *RULA: Rapid Upper Limb Assessment;
